# Succinate production positively correlates with the affinity of the global transcription factor Cra for its effector FBP in *Escherichia coli*

**DOI:** 10.1186/s13068-016-0679-7

**Published:** 2016-12-08

**Authors:** Li-Na Wei, Li-Wen Zhu, Ya-Jie Tang

**Affiliations:** Key Laboratory of Fermentation Engineering (Ministry of Education), Hubei Key Laboratory of Industrial Microbiology, Hubei Provincial Cooperative Innovation Center of Industrial Fermentation, Hubei University of Technology, Wuhan, 430068 China

**Keywords:** Succinate, Cra, Affinity, FBP, *Escherichia coli*

## Abstract

**Background:**

Effector binding is important for transcription factors, affecting both the pattern and function of transcriptional regulation to alter cell phenotype. Our previous work suggested that the affinity of the global transcription factor catabolite repressor/activator (Cra) for its effector fructose-1,6-bisphosphate (FBP) may contribute to succinate biosynthesis. To support this hypothesis, single-point and three-point mutations were proposed through the semi-rational design of Cra to improve its affinity for FBP.

**Results:**

For the first time, a positive correlation between succinate production and the affinity of Cra for FBP was revealed in *Escherichia coli*. Using the best-fit regression function, a cubic equation was used to examine and describe the relationship between succinate production and the affinity of Cra for FBP, demonstrating a significant positive correlation between the two factors (coefficient of determination *R* ^2^ = 0.894, *P* = 0.000 < 0.01). The optimal mutant strain was Tang1683, which provided the lowest mutation energy of −4.78 kcal/mol and the highest succinate concentration of 92.7 g/L, which was 34% higher than that obtained using an empty vector control. The parameters for the interaction between Cra and DNA showed that Cra bound to the promoter regions of *pck* and *aceB* to activate the corresponding genes. Normally, Cra-regulated operons under positive control are deactivated in the presence of FBP. Therefore, theoretically, the enhanced affinity of Cra for FBP will inhibit the activation of *pck* and *aceB*. However, the activation of genes involved in CO_2_ fixation and the glyoxylate pathway was further improved by the Cra mutant, ultimately contributing to succinate biosynthesis.

**Conclusions:**

Enhanced binding of Cra to FBP or active site mutations may eliminate the repressive effect caused by FBP, thus leading to increased activation of genes associated with succinate biosynthesis in the Cra mutant. This work demonstrates an important transcriptional regulation strategy in the metabolic engineering of succinate production and provides useful information for better understanding of the regulatory mechanisms of transcription factors.

**Electronic supplementary material:**

The online version of this article (doi:10.1186/s13068-016-0679-7) contains supplementary material, which is available to authorized users.

## Background

*Escherichia coli* has been widely engineered for succinate production with high titers through the inactivation or overexpression of key genes associated with succinate biosynthesis [1–3]. However, due to the complexity of the metabolic network, occasionally the desired cell phenotype may not be obtained through gene manipulation. Global regulation has attracted increasing attention because of its effective regulation of the expression of multiple pathway-related genes [4] and required cell phenotypes [5]. Furthermore, transcriptional engineering approaches have been regarded as important [6, 7]. In prokaryotes, gene expression is controlled by transcription factors (TFs) via their binding to specific DNA sequences [8]. TFs are important components of gene regulatory networks and respond to changes in the cellular environment by altering the expression of relevant genes [9]. Catabolic repressor/activator (Cra), also known as FruR, is a global TF [10, 11]. It is a dual regulator that controls the expression of a large number of metabolic genes that encode enzymes associated with the gluconeogenic pathway [12], the Krebs cycle and glyoxylate shunt [13], the Entner Doudoroff pathway [14], and the glycolytic pathway [15, 16].

As a member of the GalR-LacI family, Cra comprises two functional domains: the N-terminal DNA-binding domain and the C-terminal regulatory domain [17, 18]. The regulatory domain affects the regulatory function of Cra by altering the affinity of its DNA-binding domains for DNA upon effector binding [19]. In many TFs, effector binding has major consequences on the protein structure and function [20–22]. The effects of Cra on transcription via binding to the promoter regions of its target genes can be counteracted by its effectors, including fructose-1-phosphate (F1P) and fructose-1,6-bisphosphate (FBP) [15, 19]. In our previous work, a Cra mutant strain with 22.8% higher succinate production was obtained through random mutation and screening. Correspondingly, a higher Cra-FBP affinity (*K*_*d*_ = 130 ± 37 nM) was obtained, while the *K*_*d*_ value of wild-type Cra with FBP was 1400 ± 150 nM. It suggested that the enhanced binding affinity of the Cra mutant for FBP may contribute to succinate biosynthesis [23]. However, research on how changes in effector binding affinity modulate DNA-binding and, therefore, the cell phenotype, is limited.

To further clarify the mechanism of Cra in transcriptional regulation through effector binding and to support the hypothesis on the relationship between succinate production and the affinity of Cra for FBP, a strategy to achieve high binding affinity based on a semi-rational design was developed. A best-fit approach was then used to select regression models to best describe the relationship between succinate production and binding affinity. Finally, the regulatory effects of the optimal Cra mutants on genes associated with succinate biosynthesis and properties of the effector-Cra-DNA complexes were investigated. This work provides useful information regarding the transcriptional regulation of target product biosynthesis and represents a powerful approach for the production of other industrially important chemicals.

## Methods

### Strains, plasmids, and chemicals

All strains and plasmids used in this study are listed in Additional file 1. Primers are summarized in Additional file 2. During strain construction, the strains were cultured aerobically at 37 °C in Luria broth (5 g/L yeast extract, 10 g/L tryptone, and 10 g/L NaCl). The *E. coli* strain DH5α was used for plasmid construction. *E. coli* BL21 cells were used for the expression and purification of Cra. *E. coli* AFP111 was kindly provided by Dr. David P. Clark at Southern Illinois University [24]. The plasmids pTrc99A and pET28a were used as foundation plasmids to prepare constructs and for overexpression. rTaq DNA polymerase and primeSTAR HS DNA polymerase were purchased from Takara (Takara, Dalian, China). Restriction enzymes and T4 DNA ligase were obtained from New England Biolabs (Ipswich, USA), while the Cycle-Pure Kit, the Gel Extraction Kit, the Bacteria RNA Kit, and the Plasmids Mini Kit were obtained from Omega (Omega Bio-Tek, Doraville, USA).

### Protein structure validation and active site prediction

Crystal structures were downloaded from the RSCB Protein Data Bank (PDB) database under the following PDB ID: 2IKS (effector-free Cra from *E. coli*). The molecular models of FBP were prepared and energy-minimized in the Avogadro 1.0.2 package. Individual chains of Cra structures were aligned to chain A of the 2IKS structure using Discovery Studio (DS) 4.0.

Energy minimization of the Cra protein structure was performed by applying the “prepare protein” protocol of DS. Binding active sites were predicted using the “define and edit binding site” protocol of DS.

### Molecular docking calculations

The general docking protocol was performed as previously described [25]. Possible binding models between Cra and FBP were examined using the CDOCKER protocol of DS 4.0. The algorithm employs CHARMm force fields. A site sphere radius of 10 Å was set to assign the binding pocket.

### Computer-assisted virtual mutation

Computer-assisted virtual mutation was used to select mutations to improve the binding affinity of Cra for FBP. The amino acid residues at the active site were replaced with 17 common amino acids using the calculate mutation energy/stability module of DS 4.0. Mutation energy was the only standard used to evaluate the effect of the mutations on protein stability. The optimal mutants with the lower mutation energy, representing the higher binding affinity, were selected. The mutation energy was calculated as the sum of van der Waals, electrostatic, non-polar, and entropy terms. The single-point and three-point mutations were generated using overlap extension PCR.

### Anaerobic bottles culture

The pre-culture and fermentation medium consisted of the following components (g/L): glucose 40, yeast extract 10, tryptone 20, K_2_HPO_4_·3H_2_O 0.9, KH_2_PO_4_ 1.14, CaCl_2_· 3H_2_O 0.25, (NH4)_2_SO_4_ 3.0, MgSO_4_·7H_2_O 0.5, and VB_1_ 1 mg. For the first preculture, fresh colonies were picked from LB agar plates supplemented with ampicillin (100 mg/L), chloramphenicol (34 mg/L), and kanamycin (34 mg/L). Medium (50 mL) was prepared in a 250-mL flask, and a colony from a plate culture was inoculated and then incubated for 10 h at 37 °C on a rotary shaker at 180 rpm. For the second pre-culture, 50 mL of medium was prepared in a 250-mL rotary shaker, inoculated with 200 μL of the first pre-culture broth and incubated for 10 h at 37 °C on a rotary shaker at 180 rpm. For fermentation, 5% (v/v) of the second pre-culture was inoculated into anaerobic bottles containing 50 mL fermentation medium. After aerobic growth at 37 °C and shaking at 180 rpm for 4 h, 40 g/L MgCO_3_ was added, and a small amount of CO_2_ was vented. Cells were then transferred to anaerobic conditions by rubber plug. Samples were obtained after 96 h, and three replicates were performed in parallel.

### Fed-batch culture

Details of the preculture medium and preculture conditions have been previously described [26]. Dual-phase fed-batch fermentation was conducted with 5 L of initial fermentation medium in a 7.5-L BioFlo 115 fermenter (New Brunswick Scientific, USA). A 5% (v v^−1^) inoculums from the second preculture was used. During aerobic fermentation, the initial sugar concentration was 35 g/L. The dissolved oxygen (DO, approximately 40%) was controlled by adjusting the agitation speed and aeration rate. When the residual sugar concentration was less than 1 g/L, the aerobically grown cells were directly transferred to anaerobic conditions, and the glucose concentration was adjusted to 40 g/L by supplying glucose as an 800 g/L solution. A rotation speed of 400 rpm and 0.1 vvm of external CO_2_ gas were used. The pH was maintained at 7.0 using 20 g/L MgCO_3_ and 5 M NaOH. In the subsequent fermentation process, when the residual sugar concentration fell to less than 10 g/L, glucose was added to achieve 40 g/L. Cell mass and measurements of the residual sugar and succinate concentrations were determined as previously reported [26].

Six cultures were carried out simultaneously in stirred-tank bioreactors containing different engineered strains under identical experimental conditions; this method ensured that accurate head-to-head comparisons could be made. The results presented here were reproducible in another experiment (data not shown).

### RT-qPCR

*E. coli* AFP111 cells transformed with plasmids were collected at 10 h (the onset of anaerobic culture) during the dual-phase fed-batch fermentation, and total RNA was extracted using a Bacterial RNA Kit (Omega Bio-Tek, Doraville, USA). Total RNA fragments were reverse-transcribed into cDNA using the PrimeScript™ RT Reagent Kit (Takara, Dalian, China). And 16S rRNA was used as the endogenous control. All cDNA samples were diluted to a final concentration of 10 ng/µL. A two-step RT-PCR kit with SYBR Green was used with a thermal cycler (iCycler, Bio-Rad, USA) for RT-qPCR. Primers were used at final concentrations of 0.2 µM, and 10 ng of cDNA was used as a template in each 20 µL reaction. The threshold cycles for each sample were calculated based on fluorescence data using proprietary software (Bio-Rad, USA).

### Enzyme activity

To measure enzyme activity, cells were harvested at the onset of anaerobic culture (10 h) (i.e., at the end of the aerobic phase) during fermentation via centrifugation (12,000×*g* at 4 °C). Cells were resuspended in 50 mM potassium phosphate buffer (pH 7.4) and disrupted on ice for 15 min (with a working period of 3 s and a 5-s interval constituting each cycle) at a power output of 200 W using an ultrasonic disruptor (J92-II, Xinzhi, Ningbo, China). Unbroken cells were removed by centrifugation at 10,000×*g* for 20 min, and the supernatant was further centrifuged at 10,000×*g* for 10 min. The resulting supernatant was used in the enzyme activity assays. Enzyme activities were measured using a spectrophotometer (UV-1100, MAPADA, Shanghai, China).

All protocols used for the enzyme activity assays have been described in previous studies and were optimized for the conditions and media used in this study. Phosphofructokinase (PFK) [27], phosphoenolpyruvate carboxylase (PPC) [28], phosphoenolpyruvate carboxykinase (PCK) [28], malate dehydrogenase (MDH) [29], fumarase (FUM) [30], fumarate reductase (FRD) [28], malate synthetase (MS) [31], isocitrate lyase (ICL) [28], citrate synthetase (CS) [32], and isocitrate dehydrogenase (ICD) [33] were measured. The wavelength used to determine NAD^+^, NADH, NADP^+^, and NADPH were 340 nm, respectively. Fumarate and malate formation were recorded at 240 and 578 nm, respectively. One unit (U) of enzyme activity represents the amount of enzyme required to catalyze the conversion of 1 µmol of substrate per min into specific products. The total protein concentration in the crude cell extracts was measured using a BCA Protein Assay Kit (Beyotime, China). Enzyme assays were performed in triplicate, and if the discrepancy between the results was greater than 10%, another pair of assays was performed.

### Cloning, expression, and protein purification

The DNA sequence encoding the Cra protein was amplified from *E. coli* AFP111 using the primers Cra-*Sac*I-F and Cra-His-*Hind*III-R. To aid in the purification of Cra, a His-tag-encoding fragment was included in the construct. The resulting PCR product was digested with *Sac*I/*Hind*III and cloned into the expression vector pET28a. The resulting plasmid (pET-*cra*) was transformed into *E. coli* BL21 cells. For the expression of the His_6_-tagged protein, the transformed strain was grown in LB medium at 37 °C to an OD_600_ = 1.0. Gene overexpression was induced by the addition of 1 µM isopropyl-β-*D*-thiogalactopyranoside (IPTG) (Biosharp, Seoul, Korea). Cells were cultured overnight.

The cells were then centrifuged at 4600×*g* for 15 min, and the pellets resuspended in phosphate-buffered saline (PBS) (pH 7.4), after which time the cells were disrupted on ice using an ultrasonic disruptor (J92-II, Xinzhi, Ningbo, China). Unbroken cells were removed by centrifugation at 10,000×*g* for 30 min. Finally, the proteins were resuspended in 100 mM Tris buffer (pH 6.8) (ANGUS, USA) containing 10% β-mercaptoethanol (AMRESCO, USA), and the suspension was stored at −80 °C. For purification, the native His_6_-Cra protein was isolated from the cell lysate supernatant using Ni metal affinity resin (Clontech, USA) according to the manufacturer’s instructions. The purified protein was stored in elution buffer at 4 °C until use, as indicated in each case.

### Non-radioactive electrophoretic mobility shift assay (EMSA)

EMSA was performed as previously described [34]. The 18-bp DNA fragment used for EMSA was prepared by heating a 50 nM mixture of complementary oligonucleotides containing the presumed Cra binding site of the *pck* and *aceB* promoter regions in 1 mL TE buffer at 95 °C for 10 min. The annealed product was maintained on ice until use. Cra was incubated with the DNA fragment in the presence of 1 mM FBP (Sigma-Aldrich, 98% purity). Reaction mixtures were incubated for 15 min at 30 °C and electrophoresed on a non-denaturing 10% (w/v) polyacrylamide gel. The gels were stained with ethidium bromide and visualized using a gel imaging system (Tanon-1600, China).

### Isothermal titration microcalorimetry (ITC)

ITC experiments were performed using a TAM III instrument (Q Series Thermal Analysis) at 25 °C. Cra was thoroughly desalted using PD-10 desalting columns (GE, USA). The protein concentration of the desalted solution was determined using a BCA protein assay kit. Each ITC titration involved 10 μL injections of 50 mM FBP into a 15 μM protein solution that was placed into the 1.4-mL chamber of the apparatus. Δ*H* (reaction enthalpy), *K*_A_ (binding constant), and n (reaction stoichiometry) were determined based on the fitted curves. The changes in free energy (Δ*G*) and entropy (Δ*S*) were calculated using the following equation: Δ*G* = −*RT*ln*K*_A_ = Δ*H* − *T*Δ*S*, where *R* is the universal molar gas constant and *T* is the absolute temperature.

### Statistical analyses

The data were analyzed using IBM SPSS 19 (SPSS Inc., Chicago, IL). *P* values less than 0.05 were considered significant.

## Results and discussion

### Docking of Cra and FBP to identify the active sites for improved affinity

**Fig. 1:**
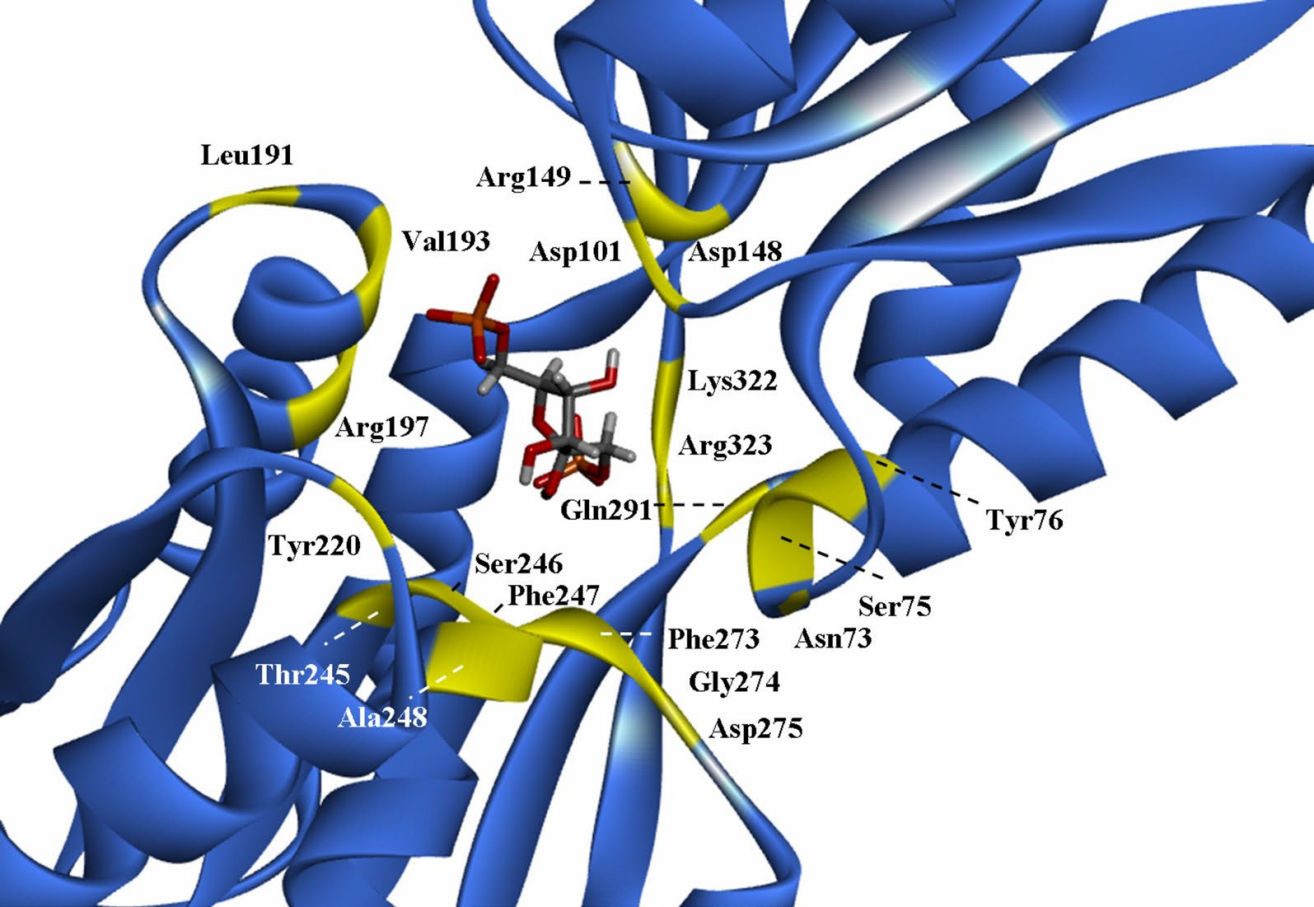
Crystal structure of Cra (PDB: 2IKS) and the docked model of Cra with FBP

To determine whether the enhanced binding affinity of each Cra mutant for its effector promotes succinate biosynthesis, a semi-rational strategy based on computer-assisted virtual saturation mutagenesis was devised to enhance the affinity of Cra for FBP. Due to the lack of complete crystal structure information on Cra, the structure of the regulatory domain (PDB ID: 2IKS) was utilized to model the effector-binding pocket by computational docking algorithms. The docked position was compared to the crystal structure of the regulatory domain by calculating root mean square deviation (RMSD) values. As shown in Fig. 1, 4 amino acids that directly interacted with FBP (Asp148, Arg149, Arg197, and Arg323) and 16 amino acids that indirectly interacted with FBP (Asn73, Ser75, Tyr76, Asp101, Leu191, Val193, Tyr220, Thr245, Ser246, Phe247, Ala248, Phe273, Gly274, Asp275, Gln291, and Lys322) were identified. These amino acids may play a significant role in the affinity of Cra for FBP. Thus, these 20 amino acids were selected as possible sites for mutagenesis.

### Correlations between succinate production and the affinity of Cra for FBP

Computer-assisted virtual saturation mutagenesis was carried out on this group of mutants to determine the optimal single-point mutants using the mutation energy as the unique standard to evaluate mutant stability and affinity. A negative mutation energy indicated that the mutation would enhance the interaction between Cra and FBP. Based on the assumption that the lower mutation energy represents the more stable protein structure and higher binding affinity, 37 single-point mutant strains were selected from more than 300 virtual mutant strains (Table 1), including 29 mutant strains (Tang1646-Tang1674) with lower mutation energy. To more accurately fit the relationship between affinity and succinate production, 8 mutation strains (Tang1675-Tang1682) with higher mutation energy were also selected.

**Table 1:** The characteristic of single-point mutations, mutation energy, and succinate production

Strain	Residue	Mutated to	Mutation energy (kcal/mol)	Effect	Succinate production (g/L)
Tang1646	Asp148	Arg	−3.15	Stabilizing	33.8
Tang1647	Gly274	Arg	−3.04	Stabilizing	33.2
Tang1648	Asp101	Arg	−2.84	Stabilizing	36.7
Tang1649	Ser75	Lys	−2.60	Stabilizing	35.0
Tang1650	Tyr220	Lys	−2.40	Stabilizing	32.7
Tang1651	Asp148	Lys	−2.34	Stabilizing	34.8
Tang1652	Leu191	Lys	−2.04	Stabilizing	34.2
Tang1653	Asn73	Tyr	−1.92	Stabilizing	32.2
Tang1654	Ser246	Lys	−1.90	Stabilizing	32.1
Tang1655	Phe273	Lys	−1.58	Stabilizing	31.6
Tang1656	Tyr76	Arg	−1.51	Stabilizing	31.6
Tang1657	Asp275	Glu	−1.50	Stabilizing	32.5
Tang1658	Gln291	Arg	−1.32	Stabilizing	32.2
Tang1659	Ala248	Lys	−1.22	Stabilizing	33.9
Tang1660	Arg323	Lys	−1.06	Stabilizing	31.7
Tang1661	Asn73	Arg	−1.05	Stabilizing	32.6
Tang1662	Ser75	Arg	−1.04	Stabilizing	31.3
Tang1663	Thr245	Ser	−1.04	Stabilizing	30.6
Tang1664	Asp275	Gln	−1.02	Stabilizing	30.5
Tang1665	Asn73	Phe	−0.84	Stabilizing	31.8
Tang1666	Lys322	Asp	−0.84	Stabilizing	32.7
Tang1667	Phe247	Pro	−0.81	Stabilizing	32.4
Tang1668	Thr74	Lys	−0.75	Stabilizing	32.0
Tang1669	Ala248	Phe	−0.72	Stabilizing	30.5
Tang1670	Arg149	Lys	−0.67	Stabilizing	31.4
Tang1671	Asp148	Glu	−0.40	Destabilizing	33.1
Tang1672	Tyr220	Pro	−0.22	Destabilizing	32.0
Tang1673	Tyr220	Leu	−0.20	Destabilizing	29.0
Tang1674	Arg197	Lys	−0.04	Destabilizing	30.4
Tang1675	Phe247	Glu	0.38	Destabilizing	31.3
Tang1676	Tyr76	Asp	0.95	Destabilizing	30.1
Tang1677	Phe247	Arg	1.08	Destabilizing	29.3
Tang1678	Ser75	Glu	1.65	Destabilizing	27.0
Tang1679	Arg149	Phe	1.65	Destabilizing	29.8
Tang1680	Arg149	His	2.24	Destabilizing	27.2
Tang1681	Gln291	Met	2.54	Destabilizing	25.7
Tang1682	Gln291	Trp	3.57	Destabilizing	24.3

**Fig. 2:**
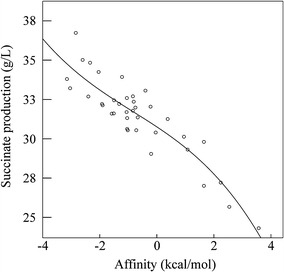
Correlation between succinate production by single-point mutant strains and the affinity of the Cra mutants for FBP

The effects of single-point Cra mutations on succinate production are shown in Table 1. To describe the relationship between succinate production and the affinity of Cra for FBP, a best-fit approach was used to select regression models—considering linear, logarithmic, quadratic, cubic, power, and exponential methods. The cubic regression model was chosen as the best model to determine the correlation between the binding affinity and succinate production. As shown in Fig. 2 and Additional file 3, within the mutation energy range examined, succinate production increased with increasing mutation energy (coefficient of determination *R* ^*2*^ = 0.894, *P* = 0.000 < 0.01). These results suggested a significant positive correlation between the two variables within a certain range.

**Fig. 3:**
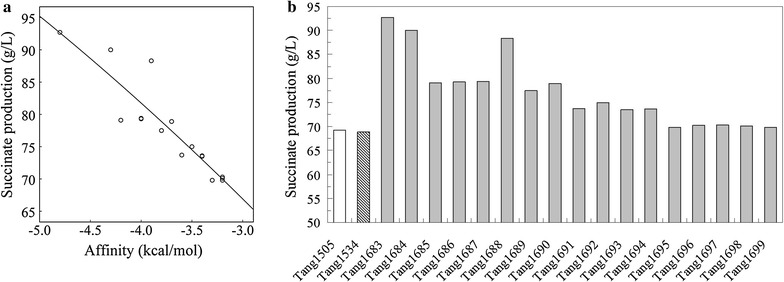
Correlation between succinate production by three-point mutant strains and affinity of the Cra mutants for FBP (**a**). Succinate production by the wild-type and three-point mutants in a bioreactor (**b**)

Because the increased binding affinity caused by single-point mutation was limited, three-point mutations were designed using the *Calculate Mutation Energy/Stability* module of DS 4.0 to provide increased affinity beyond what could be obtained by incorporating a single mutation. Based on the above results, all 37 single-point mutation sites were combined to form three-point mutations. The optimal three-point mutants were selected, in which mutation energy were lower than the lowest energy in single-point mutation. Finally, 17 optimal three-point mutant strains were generated (Table 2). Due to the extension of the affinity range, a fitting analysis of mutation energy data and succinate production was conducted using the cubic regression model. As shown in Fig. 3a, a high correlation between the mutation energies of the three-point mutants and succinate production was revealed (coefficient of determination *R* ^*2*^ = 0.840, *P* = 0.000 < 0.01), proving that the affinity of the Cra mutant closely reflects the level of succinate production. Thus, succinate production improves with increased binding affinity.

**Table 2:** The characteristic of three-point mutations, mutation energy, and succinate production

Strain	Mutated to	Mutation energy (kcal/mol)	Succinate production (g/L)
Tang1683	Asp101Arg + Asp148Arg + Gly274Arg	−4.78	92.7
Tang1684	Asp148Arg + Ala248Phe + Gly274Arg	−4.29	90.0
Tang1685	Asp148Arg + Tyr220Lys + Gly274Arg	−4.19	79.1
Tang1686	Asp101Arg + Asp148Arg + Ser246Lys	−4.03	79.3
Tang1687	Asp148Arg + Gly274Arg + Asp275Gln	−3.99	79.4
Tang1688	Asn73Tyr + Asp148Arg + Gly274Arg	−3.89	88.3
Tang1689	Ser75Lys + Ala248Phe + Gly274Arg	−3.75	77.5
Tang1690	Asp148Arg + Thr245Ser + Gly274Arg	−3.68	78.9
Tang1691	Asp148Arg + Ser246Lys + Gly274Arg	−3.63	73.7
Tang1692	Ser75Lys + Ser246Lys + Gly274Arg	−3.45	75.0
Tang1693	Asp148Arg + Ser246Lys + Phe247Pro	−3.36	73.5
Tang1694	Asp148Arg + Phe273Lys + Gly274Arg	−3.36	73.6
Tang1695	Asp148Arg + Tyr220Lys + Ser246Lys	−3.33	69.8
Tang1696	Asp148Arg + Phe247Pro + Gly274Arg	−3.22	70.3
Tang1697	Asp148Arg + Ser246Lys + Ala248Phe	−3.18	70.3
Tang1698	Ser75Lys + Phe273Lys + Gly274Arg	−3.17	70.1
Tang1699	Ser75Lys + Gly274Arg + Asp275Gln	−3.16	69.8

Figure 3b shows the succinate production of the 17 three-point mutant strains in a 7.5-L bioreactor. Time profiles of the fed-batch culture are summarized in Additional file 4. Increased succinate production was obtained with Tang1683 (Asp101Arg/Asp148Arg/Gly274Arg), Tang1684 (Asp148Arg/AlaA248Phe/Gly274Arg), and Tang 1688 (Asn73Tyr/Asp148Arg/Gly274Arg). The levels were 34, 30, and 28% higher, respectively, than that obtained with empty vector control Tang1509. These 3 optimal mutant strains were selected for further analysis.

### Structural basis for the improved affinity

**Fig. 4:**
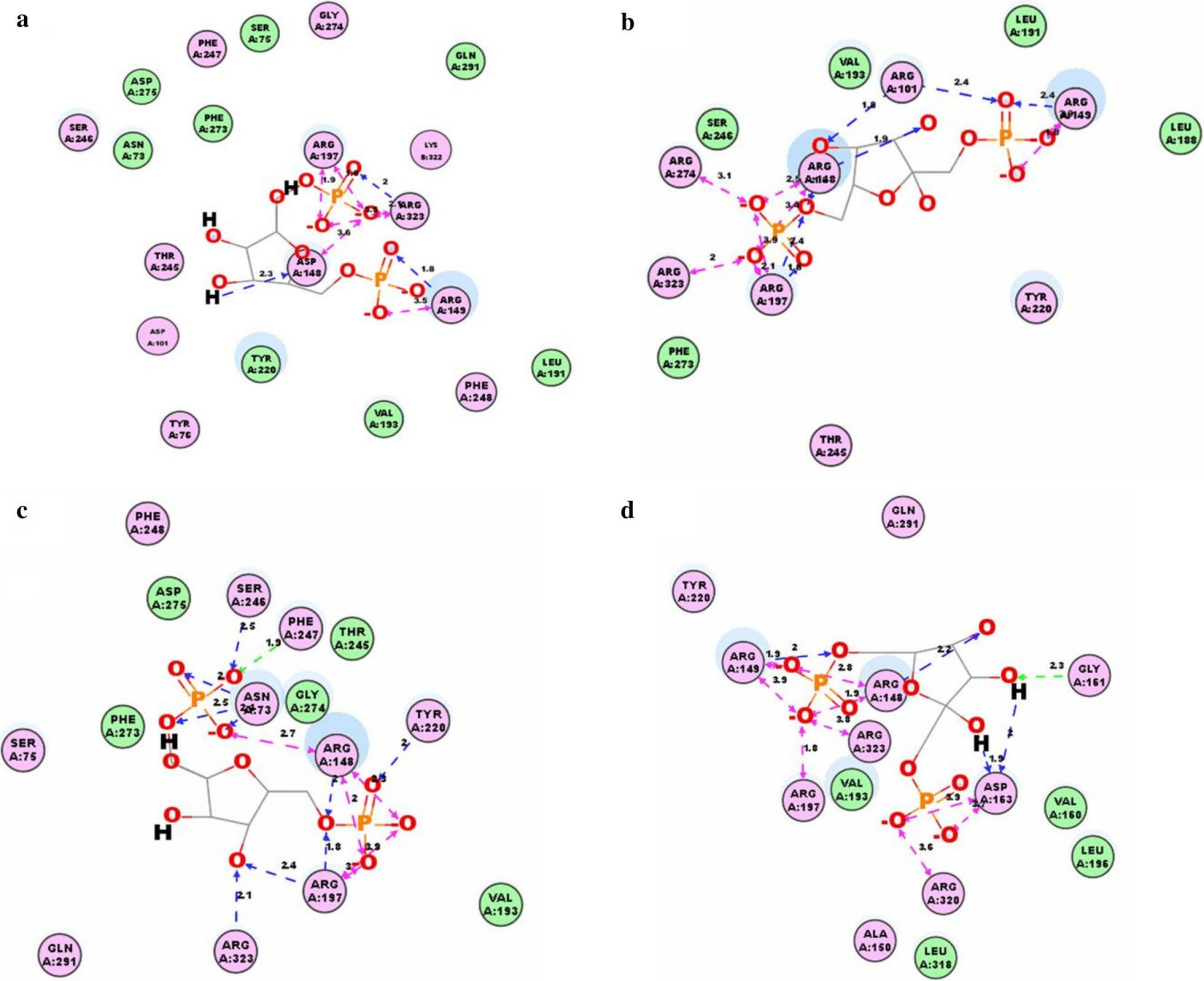
Predicted interaction between Cra and FBP in Tang1534 (**a**), Tang1683 (**b**), Tang1684 (**c**), and Tang1688 (**d**). *Blue* and *green arrows* indicate hydrogen bonds, and *pink arrows* indicate electrostatic interactions

To investigate the mechanism responsible for the improved affinity, the structures of the wild-type Cra and the optimal three-point mutants were compared. As shown in Fig. 4a, three hydrogen bonds and five electrostatic interactions were observed in the wild-type Cra model. The Cra mutations in Tang1683 resulted in four additional hydrogen bonds involving amino acids Asp101 and Arg197 and two additional electrostatic interactions (Fig. 4b). The Cra mutations in Tang1684 resulted in seven additional hydrogen bonds, while two additional hydrogen bonds and four additional electrostatic interactions were observed in the Cra mutant Tang1688 (Fig. 4c, d). The additional interaction forces may have led to closer binding between Cra and FBP. The energy changes in the Cra-FBP binding complexes were 4.78, 4.29, and 3.89 kJ/mol in Tang1683, Tang1684, and Tang1688, respectively (Table 2).

**Fig. 5:**
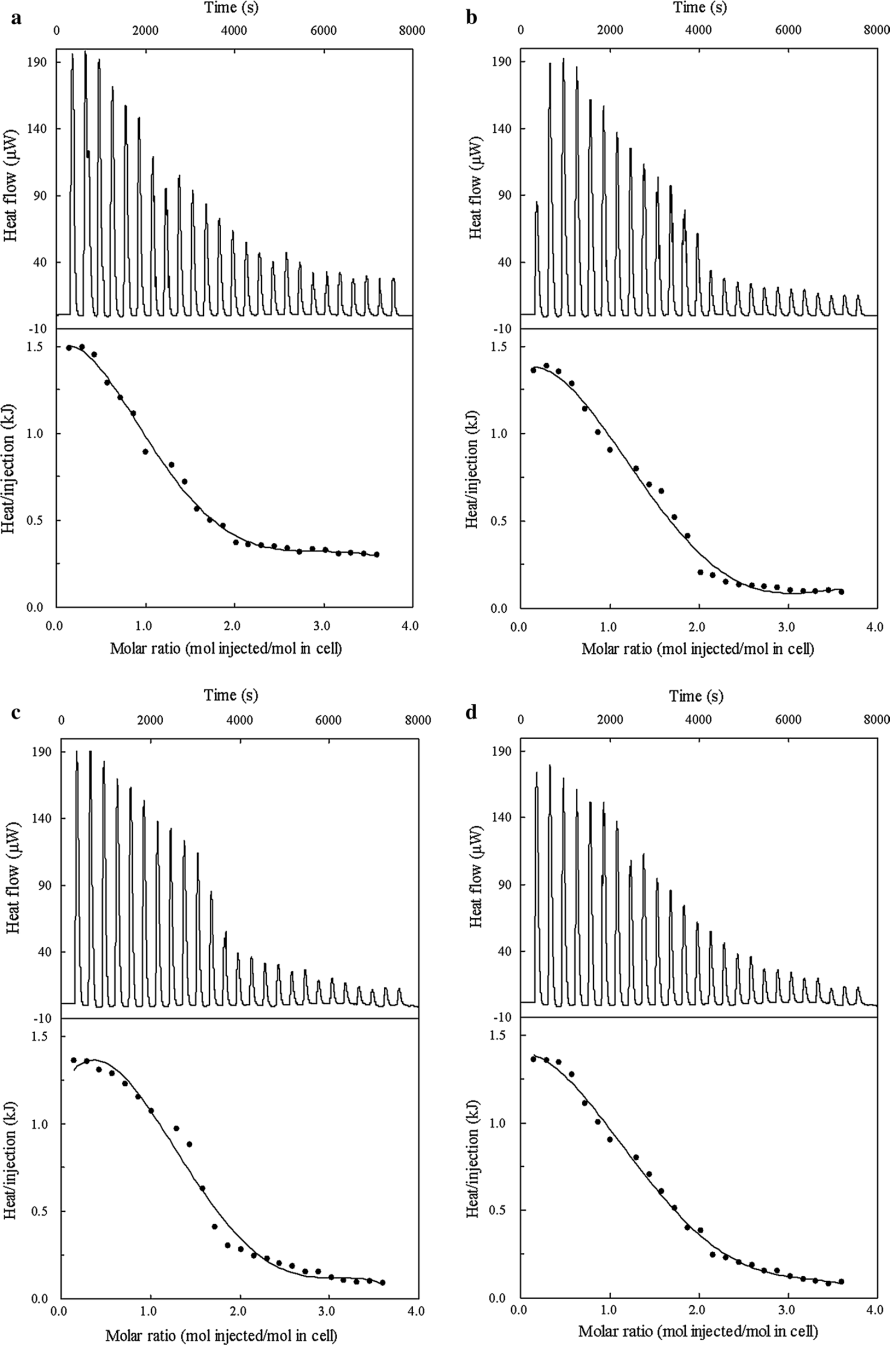
ITC assays with Cra and FBP. For titration, Cra at the concentration of 15 mM was introduced into sample cells; the ligand concentration in buffer was 50 mM. The titration of FBP with Cra of Tang1534 (**a**), Tang1683 (**b**), Tang1684 (**c**), and Tang1688 (**d**)

To determine whether the affinities of the Cra mutants for FBP were enhanced, the interaction parameters of the Cra protein with FBP were examined by ITC. As shown in Fig. 5a, the binding of wild-type Cra to FBP was an exothermic process (Δ*H* = −27.4 ± 1.3 MJ/mol, Δ*S* = −54.8 ± 0.8 kJ/mol, Δ*G* = −16.8 ± 1.4 kJ/mol). Subsequent calculations allowed us to determine a Cra-FBP dissociation constant (*K*_*d*_) of 1360 ± 6.2 nM, reflecting an interaction between FBP and wild-type Cra. The results shown in Fig. 5b indicate that the binding of FBP to the Cra mutant (Tang1683) was enthalpy-driven (Δ*H* = −10.2 ± 0.7 MJ/mol) and produced a total free energy change (Δ*G* = −18.8 ± 1.2 kJ/mol). Favorable (negative) enthalpy changes can be attributed to hydrogen bonding or a van der Waals force interaction between FBP and amino acids at the effector-binding site. Further calculations revealed a high protein-effector affinity (*K*_*d*_ = 154.2 ± 4.3 nM). The interaction results for the Tang1684 Cra mutant with FBP were similar to those of Tang1683 (Fig. 5c). The interaction was both enthalpy-driven (Δ*H* = −21.0 ± 1.3 MJ/mol) and entropy-driven (Δ*S* = 54.7 ± 2.3 kJ/mol), producing a total free energy change (Δ*G* = −16.3 ± 0.7 kJ/mol). Moreover, the protein-effector affinity was high (*K*_*d*_ = 728.3 ± 3.8 nM). The interaction results for the Tang1688 Cra mutant with FBP are shown in Fig. 5d. The interaction was enthalpy-driven (Δ*H* = −16.3 ± 0.8 MJ/mol), producing a total free energy change (Δ*G* = −23.4 ± 0.4 kJ/mol). A high protein-effector affinity was also observed (*K*_*d*_ = 809.1 ± 4.6 nM), indicating that the mutation enhanced the affinity of Cra for FBP and suggesting that the improved succinate production by the Cra mutant is likely associated with the improved affinity of Cra for FBP.

### The expression levels of genes and activities of enzymes involved in succinate biosynthesis are affected by Cra mutations

To explore the effects of Cra mutations on succinate biosynthesis, first, the mRNA transcripts from 11 genes involved in succinate biosynthesis were analyzed using semi-quantitative RT-qPCR. The activities of 10 enzymes were then measured during the fermentation period. The 11 genes, including *pfkB* (encoding phosphofructokinase, PFK), *ppc* (encoding phosphoenolpyruvate carboxylase, PPC), *pck* (encoding phosphoenolpyruvate carboxykinase, PCK), *mdh* (encoding malate dehydrogenase, MDH), *fumB* (encoding fumarase, FRD), *frdA* (encoding fumarate reductase, FUM), *gltA* (encoding citrate synthetase, CS), *icd* (encoding isocitrate dehydrogenase, ICDH), *aceA* (encoding isocitrate lyase, ICL), *aceB* (encoding malate synthetase, MS), and *iclR* (encoding IclR transcriptional repressor), and 10 corresponding enzymes were selected based on their association with succinate biosynthesis.

**Fig. 6:**
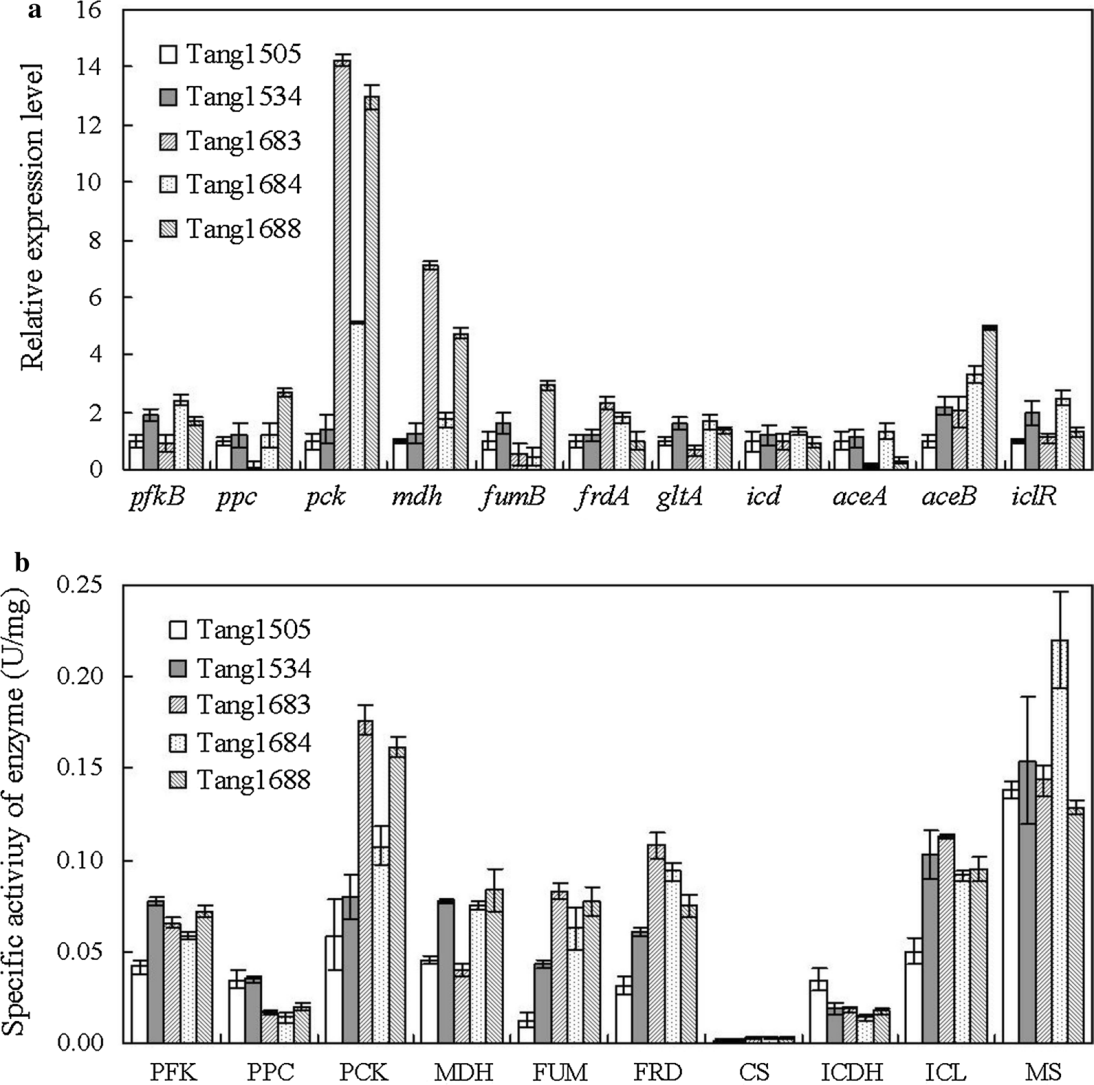
Effect of Cra mutation on the relative expression levels of genes (**a**) and the activities of enzymes (**b**) involved in succinate biosynthesis. Central metabolism of *E. coli*. *PFK* phosphofructokinase, *PPC* phosphoenolpyruvate carboxylase, *PCK* phosphoenolpyruvate carboxykinase, *MDH* malate dehydrogenase, *FUM* fumarate, *FRD* fumarate reductase, *CS* citrate synthase, *ICDH* isocitrate dehydrogenase, *ICL* isocitrate lyase, *MS* malate synthetase. Experiments were conducted in triplicate, and the *error bars* represent the standard deviation

As shown in Fig. 6a, RT-qPCR revealed that *pck* and *aceB* were significantly up-regulated in Tang1683, Tang1684, and Tang1688. Cra acts as an activator of genes involved in the glyoxylate shunt [13] and *pck* involved in phosphoenolpyruvate (PEP) carboxylation [16]. After mutating Cra, this activation was enhanced, confirming that the glyoxylate shunt and PEP carboxylation are important pathways for succinate biosynthesis [26, 35]. These obvious positive transcriptional influences may promote the biosynthesis of succinate. It should be noted that *ppc* was up-regulated in Tang1688 and that *aceA* was down-regulated in Tang1683 and Tang1688, although previous studies have demonstrated that Cra inhibits *ppc* and activates *aceA* [13, 36]. These results suggest that Cra mutations may partially change its regulatory mode and effects. Mutations in Asn73 and Asp101 were particularly important for the regulatory function of Cra. However, after mutation, the expression levels of 27 genes involved in the glycolytic pathway, the sugar phosphotransferase system, and propionate catabolism, among other areas, which should be repressed by Cra, were still down-regulated (data not shown). The expression levels of genes involved in the reductive branch (*mdh*, *fumB*, and *frdA*) of the TCA cycle were partially up-regulated in Tang1683 and Tang1688. This result may be due to the interaction between Cra and other transcription factors, such as cyclic AMP receptor protein (CRP). In a previous report, Cra positively controlled the expression of the *crp* gene [37], while *mdh* and *fumB* were activated by CRP [38].

As shown in Fig. 6b, PFK activity was the highest in Tang1534. This result indicates that PFK is activated by Cra overexpression but not by its mutation. After mutating Cra, the activity of PPC decreased, while PCK activity significantly increased. The patterns of PCK activity were similar to those observed by expressing the corresponding genes, suggesting that PCK is the main enzyme catalyzing PEP carboxylation in the Cra mutants and that the obvious enhancement of gene expression and PCK activity may promote succinate biosynthesis. Active PEP carboxylation may increase the concentration of oxaloacetate (OAA), which could activate enzymes involved in the reductive TCA cycle as substrates. The activities of FUM, FRD, and CS were all improved after mutation. However, the activity of ICDH, which catalyzes the conversion of isocitrate to α-ketoglutarate, decreased after mutation, suggesting that the intermediate isocitrate is shunted by other branches such as the glyoxylate pathway. This inference was proven by the enhanced ICL activity. MS activity was the highest in the Cra mutant (Tang1684). The enhanced activities of enzymes in the glyoxylate pathway may thus promote succinate biosynthesis.

In summary, most genes and enzymes involved in PEP carboxylation, the glyoxylate pathway, and the reductive TCA cycle were activated due to the mutation of Cra either directly or indirectly, in particular *pck* and *aceB*. This activation may explain why succinate production was significantly improved after mutation. These phenotypes are similar to those reported in other global regulatory reports [4]. When *E. coli* NZN111 cells were grown aerobically on acetate, succinic acid production was greatly improved without further genetic modifications. Additionally, the activities of ICL, MDH, and PCK were greatly enhanced, suggesting that global regulation could be an effective approach by which to enhance succinate production.

### Enhanced DNA binding by Cra mutation

**Fig. 7:**
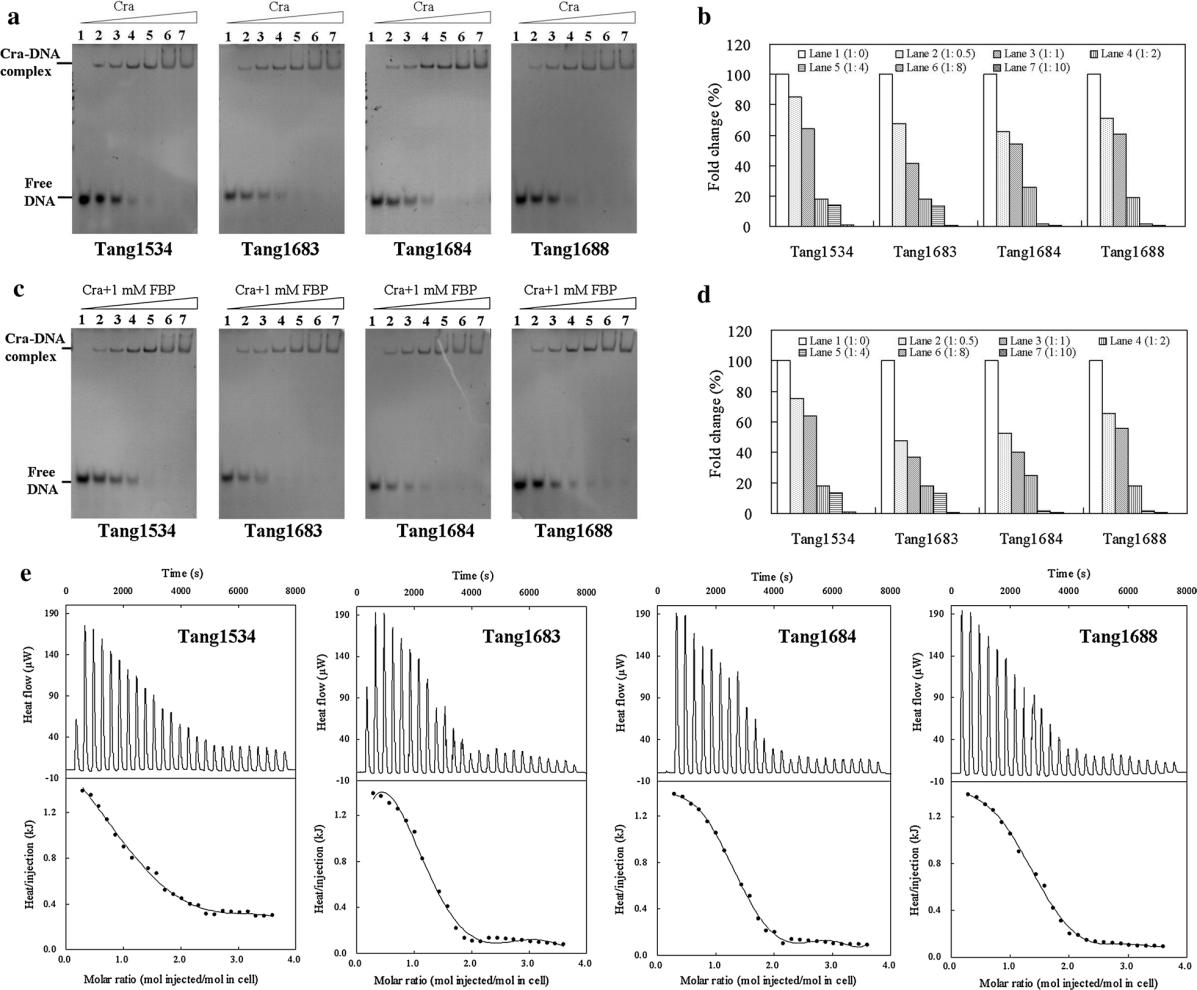
The combination of Cra and *pckp* by EMSA and ITC experiments. EMSA experiment with increasing concentrations of pure Cra protein. Molar ratios of *pckp*:Cra were 1:0 (*lane 1*), 1:0.5 (*lane 2*), 1:1 (*lane 3*), 1:2 (*lane 4*), 1:4 (*lane 5*), 1:8 (*lane 6*), and 1:10 (*lane 7*). *pckp* (1.5 μM) in the absence of FBP (**a**) and the *gray value* determined using Quantity one (**b**). *pckp* (1.5 μM) in the presence of 1 mM FBP (**c**) and the *gray value* (**d**). ITC experiment with 15 mM wild-type Cra or mutant variants binding to 1 mM *pckp* DNA with 50 mM FBP (**e**)

To verify that the activation of *pck* and *aceB* was directly caused by Cra mutation, a non-radioactive EMSA was conducted to demonstrate the binding of DNA to Cra. As shown in Fig. 7a, when Cra was present in the reaction alone, increasing amounts of Cra enhanced binding. To more clearly describe the changes in binding, a gray-scale analysis was performed (Fig. 7b). The band intensity increased with increasing Cra concentrations, and the amount of free DNA decreased with decreasing Cra concentrations. Compared with wild-type Cra, the binding of the *pck* promoter sequence to the Cra mutants was enhanced. When Cra was present with FBP, there was no significant change in the interaction between wild-type Cra and *pckp* (Fig. 7c). The binding of *pckp* to the Cra mutants was further enhanced in the presence of FBP, particularly in Tang1683 (Fig. 7d). As shown in Fig. 7e, the binding of *pckp* (Tang 1534) to Cra with FBP was an endothermic process (Δ*H* = −27.4 ± 1.3 MJ/mol, Δ*S* = −91.8 ± 1.4 kJ/mol, Δ*G* = −16.8 ± 0.5 kJ/mol). The *K*_*d*_ values of *pckp* binding to Cra in Tang1534, Tang1683, Tang1683, and Tang1688 were 1123.6 ± 4.2, 90.9 ± 0.6, 133.3 ± 1.3, and 171.0 ± 1.6 nM, respectively, despite previous reports suggesting that Cra-regulated operons under positive control would be deactivated in the presence of FBP [15, 19]. Therefore, theoretically, the enhanced interaction between Cra and FBP would inhibit the activation of *pck*. However, after Cra mutation, the activation of genes involved in CO_2_ fixation was further improved with enhanced binding affinity (Fig. 6a). This result indicates that FBP is invalid and can be explained by two possibilities: first, when the affinity of Cra for FBP increased, the close interaction affected the regulatory function of FBP, thereby eliminating the inhibitory effect of FBP on Cra, and the elimination effect was enhanced with increased binding affinity; second, mutation of the active sites may have led to changes in the structure of the binding cavities, so that FBP could not perform its normal regulatory functions by binding to Cra. In this situation, when the binding affinity of the Cra mutants for FBP was enhanced, the positive effect of Cra on the expression of genes (such as *pck*) was further strengthened, ultimately contributing to succinate biosynthesis.

**Fig. 8:**
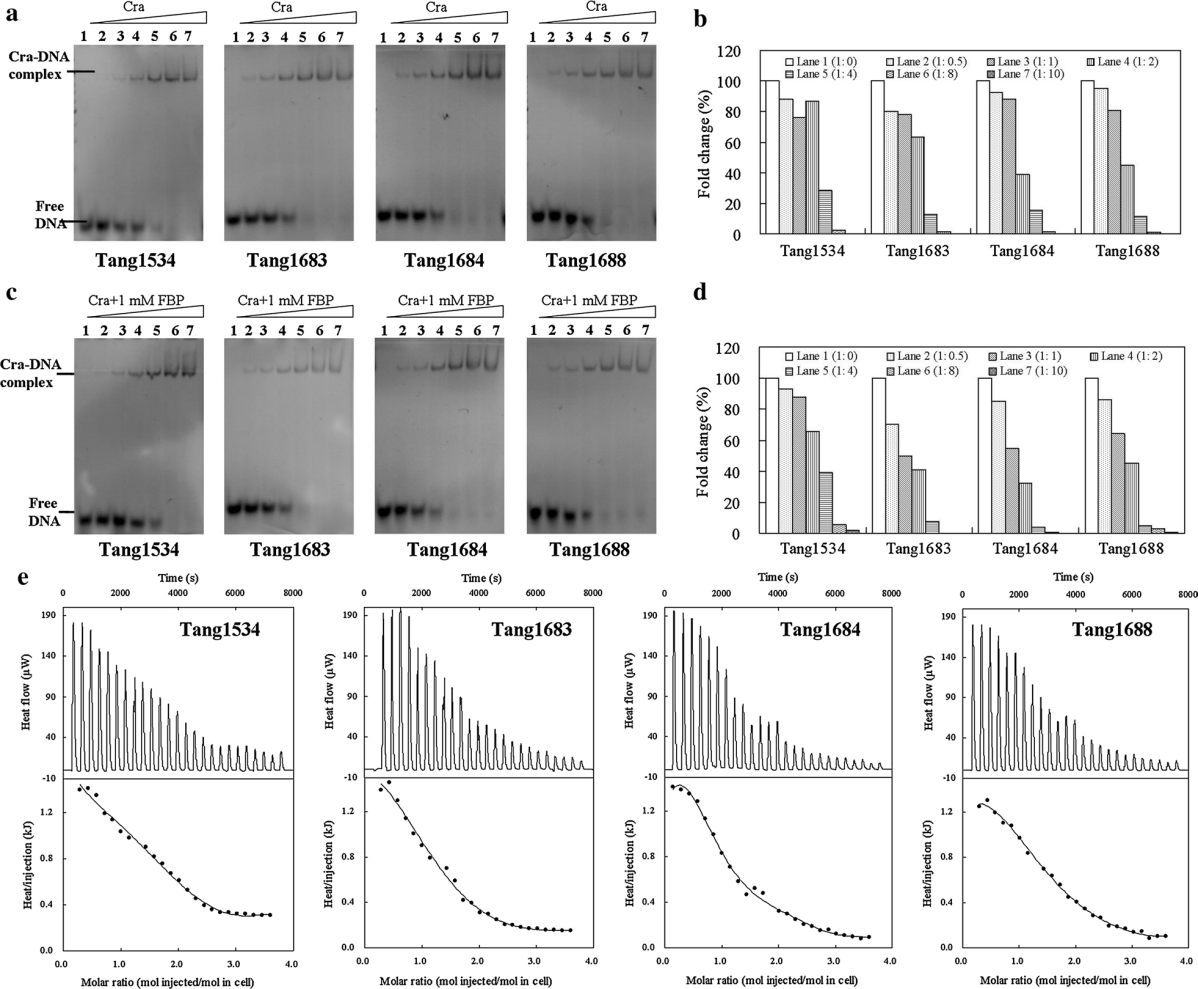
The combination of Cra and *acep* by EMSA and ITC experiments. EMSA experiment with increasing concentrations of pure Cra protein. Molar ratios of *acep*:Cra were 1:0 (*lane 1*), 1:0.5 (*lane 2*), 1:1 (*lane 3*), 1:2 (*lane 4*), 1:4 (*lane 5*), 1:8 (*lane 6*), and 1:10 (*lane 7*). *acep* (1.5 μM) in the absence of FBP (**a**) and the *gray value* determined using Quantity one (**b**). *acep* (1.5 μM) in the presence of 1 mM FBP (**c**) and the *gray value* (**d**). ITC experiment with 15 mM wild-type Cra or mutations binding to 1 mM *acep* DNA with 50 mM FBP (**e**)

The binding of *aceB* promoter sequences to Cra was similar to that of *pckp*. This set of data clearly demonstrates that compared to wild-type Cra, the binding affinity of *aceBp* to the Cra mutants was enhanced in the presence of FBP (Fig. 8c, d). However, when FBP was absent, the enhanced interaction was accompanied by the increased protein concentrations of the Cra mutants (Fig. 8a, b). The *K*_*d*_ values of *aceBp* binding to Cra with FBP in Tang1534, Tang1683, Tang1683, and Tang1688 were 1102.2 ± 4.7, 271.8 ± 1.3, 340.1 ± 1.8, and 410.0 ± 2.6 nM, respectively (Fig. 8e). The contacts between *aceBp* and Cra led to a lower affinity binding interaction than that between *pckp* and Cra. This weakened interaction was consistent with the decreased level of gene expression.

## Conclusions

To support the hypothesis regarding the relationship between succinate biosynthesis and the binding affinity of Cra for FBP, several saturation mutations aimed at improving binding affinity were generated through the semi-rational design of the Cra protein. As the most appropriate model, cubic regression was used to describe the relationship, and a positive correlation between the binding affinity of Cra for FBP and succinate production was demonstrated for the first time. The highest succinate production of 92.7 g/L was obtained using the optimal mutant strain (Tang1683) with the highest binding affinity. The inhibitory effect caused by FBP was eliminated, likely because of the enhanced binding affinity between Cra and FBP or by mutations at active sites. Most genes and enzymes involved in PEP carboxylation, the glyoxylate pathway, and the reductive TCA cycle were activated upon mutation of Cra, either directly or indirectly, especially *pck* and *aceB*, ultimately contributing to succinate biosynthesis.

## Additional files

**Additional file 1: Table S1.** Strains and plasmids used in this study.

**Additional file 2: Table S2.** Primers used in this study.

**Additional file 3: Table S3.** The relationship between succinate production and the affinity of Cra for FBP.

**Additional file 4: Figure S1.** Time courses of fed-batch cultures of the 17 three-point mutant strains in 7.5-L bioreactor.

## Abbreviations

Cra: catabolite repressor/activator
TFs: transcription factors
F1P: fructose-1-phosphate
FBP: fructose-1,6-bisphosphate
RT-qPCR: reverse transcription quantitative polymerase chain reaction
PFK: phosphofructokinase
PPC: phosphoenolpyruvate carboxylase
PCK: phosphoenolpyruvate carboxykinase
MDH: malate dehydrogenase
FUM: fumarase
FRD: fumarate reductase
MS: malate synthetase
ICL: isocitrate lyase
CS: citrate synthetase
ICDH: isocitrate dehydrogenase
ITC: isothermal titration microcalorimetry
EMSA: electrophoretic mobility shift assay
TCA: tricarboxylic acid
IclR: isocitrate lyase regulator
CRP: cyclic AMP receptor protein
