# Supplementary material for: Succinate production positively correlates with the affinity of the global transcription factor Cra for its effector FBP in Escherichia coli
Source: Biotechnol Biofuels. 2016 Dec 8;9:264. doi: 10.1186/s13068-016-0679-7 (PMC5146860; doi:10.1186/s13068-016-0679-7)
Supplement: Supplementary file 1 — Additional file 1: Table S1. Strains and plasmids used in this study. [file 13068_2016_679_MOESM1_ESM.pdf]

Table S1. Strains and plasmids used in this study.

Strains/Plasmids	Relevant characteristics	Sources or reference
**Strains**		
AFP111	F+ λ- *rpo*S396(Am) rph-1 Δ*pflAB*::Cam *ldhA*::Kan *ptsG*	[18]
DH5α	F-φ80 *lac*ZΔM15 Δ(*lac*ZYA-*arg*F) U169 *end* A1 *rec*A1 *hsd*R17(r_k-, m_k-) *sup* E44λ- *thi*-1 *gyr*A96 *rel*A1 *pho*A	TransGen Biotech
BL21(DE3)	F$^-$*omp* T*hsd*S$_B$($r_B^-m_B^-$) *gal dcm*	TransGen Biotech
Tang1505	AFP111/ pTrc99A	This study
Tang1534	AFP111/ pTrc-*cra*	This study
Tang1646	AFP111/ pTrc99A carrying the *cra* gene with mutation at D148R	This study
Tang1647	AFP111/ pTrc99A carrying the *cra* gene with mutation at G274R	This study
Tang1648	AFP111/ pTrc99A carrying the *cra* gene with mutation at D101R	This study
Tang1649	AFP111/ pTrc99A carrying the *cra* gene with mutation at S75K	This study
Tang1650	AFP111/ pTrc99A carrying the *cra* gene with mutation at Y220K	This study
Tang1651	AFP111/ pTrc99A carrying the *cra* gene with mutation at D148K	This study
Tang1652	AFP111/ pTrc99A carrying the *cra* gene with mutation at L191K	This study
Tang1653	AFP111/ pTrc99A carrying the *cra* gene with mutation at N73Y	This study
Tang1654	AFP111/ pTrc99A carrying the *cra* gene with mutation at S246K	This study
Tang1655	AFP111/ pTrc99A carrying the *cra* gene with mutation at F273K	This study
Tang1656	AFP111/ pTrc99A carrying the *cra* gene with mutation at Y76R	This study
Tang1657	AFP111/ pTrc99A carrying the *cra* gene with mutation at D275E	This study
Tang1658	AFP111/ pTrc99A carrying the *cra* gene with mutation at Q291R	This study
Tang1659	AFP111/ pTrc99A carrying the *cra* gene with mutation at A248K	This study
Tang1660	AFP111/ pTrc99A carrying the *cra* gene with mutation at R323K	This study
Tang1661	AFP111/ pTrc99A carrying the *cra* gene with mutation at N73R	This study

Tang1662	AFP111/ pTrc99A carrying the *cra* gene with mutation at S75R	This study
Tang1663	AFP111/ pTrc99A carrying the *cra* gene with mutation at T245S	This study
Tang1664	AFP111/ pTrc99A carrying the *cra* gene with mutation at D275Q	This study
Tang1665	AFP111/ pTrc99A carrying the *cra* gene with mutation at N73F	This study
Tang1666	AFP111/ pTrc99A carrying the *cra* gene with mutation at K322D	This study
Tang1667	AFP111/ pTrc99A carrying the *cra* gene with mutation at F247P	This study
Tang1668	AFP111/ pTrc99A carrying the *cra* gene with mutation at T74K	This study
Tang1669	AFP111/ pTrc99A carrying the *cra* gene with mutation at A248F	This study
Tang1670	AFP111/ pTrc99A carrying the *cra* gene with mutation at R149K	This study
Tang1671	AFP111/ pTrc99A carrying the *cra* gene with mutation at D148E	This study
Tang1672	AFP111/ pTrc99A carrying the *cra* gene with mutation at Y220P	This study
Tang1673	AFP111/ pTrc99A carrying the *cra* gene with mutation at Y220L	This study
Tang1674	AFP111/ pTrc99A carrying the *cra* gene with mutation at R197K	This study
Tang1675	AFP111/ pTrc99A carrying the *cra* gene with mutation at F247E	This study
Tang1676	AFP111/ pTrc99A carrying the *cra* gene with mutation at Y76D	This study
Tang1677	AFP111/ pTrc99A carrying the *cra* gene with mutation at F247R	This study
Tang1678	AFP111/ pTrc99A carrying the *cra* gene with mutation at S75E	This study
Tang1679	AFP111/ pTrc99A carrying the *cra* gene with mutation at R149F	This study
Tang1680	AFP111/ pTrc99A carrying the *cra* gene with mutation at R149H	This study
Tang1681	AFP111/ pTrc99A carrying the *cra* gene with mutation at Q291M	This study
Tang1682	AFP111/ pTrc99A carrying the *cra* gene with mutation at Q291W	This study
Tang1683	AFP111/ pTrc99A carrying the *cra* gene with mutation at D101R, D148R, and G274R	This study
Tang1684	AFP111/ pTrc99A carrying the *cra* gene with mutation at D148R, A248F, and G274R	This study

Tang1685	AFP111/ pTrc99A carrying the *cra* gene with mutation at D148R, Y220K, and G274R	This study
Tang1686	AFP111/ pTrc99A carrying the *cra* gene with mutation at D101R, D148R, and S246K	This study
Tang1687	AFP111/ pTrc99A carrying the *cra* gene with mutation at D148R, G274R, and D275Q	This study
Tang1688	AFP111/ pTrc99A carrying the *cra* gene with mutation at N73Y, D148R, and G274R	This study
Tang1689	AFP111/ pTrc99A carrying the *cra* gene with mutation at S75K, A248F, and G274R	This study
Tang1690	AFP111/ pTrc99A carrying the *cra* gene with mutation at D148R, T245S, and G274R	This study
Tang1691	AFP111/ pTrc99A carrying the *cra* gene with mutation at D148R, S246K, and G274R	This study
Tang1692	AFP111/ pTrc99A carrying the *cra* gene with mutation at S75K, S246K, and G274R	This study
Tang1693	AFP111/ pTrc99A carrying the *cra* gene with mutation at D148R, S246K, and F247P	This study
Tang1694	AFP111/ pTrc99A carrying the *cra* gene with mutation at D148R, F273K, and G274R	This study
Tang1695	AFP111/ pTrc99A carrying the *cra* gene with mutation at D148R, Y220K, and S246K	This study
Tang1696	AFP111/ pTrc99A carrying the *cra* gene with mutation at D148R, F247P, and G274R	This study
Tang1697	AFP111/ pTrc99A carrying the *cra* gene with mutation at D148R, S246K, and A248F	This study
Tang1698	AFP111/ pTrc99A carrying the *cra* gene with mutation at S75K, F273K, and G274R	This study
Tang1699	AFP111/ pTrc99A carrying the *cra* gene with mutation at S75K, G274R, and D275Q	This study
**Plasmids**		
pTrc99A	Amp^R, pBR322 ori, $lacI^q$, *trc* promoter	Invitrogen
pET28a	Kan^R, pBR322 ori, $lacI^q$, T7 promoter	Novagene
pTrc-*cra*	pTrc99A carrying *cra* gene	This study
pET-*cra*	pET28a carrying *cra* gene	This study
pET-CI	pET28a carrying *cra* gene with mutation at D101R, D148R, and G274R	This study
pET-CII	pET28a carrying *cra* gene with mutation at D148R, A248F, and G274R	This study
pET-CIII	pET-28a carrying *cra* gene with mutation at N73Y, D148R, and G274R	This study
