# Supplementary material for: Succinate production positively correlates with the affinity of the global transcription factor Cra for its effector FBP in Escherichia coli
Source: Biotechnol Biofuels. 2016 Dec 8;9:264. doi: 10.1186/s13068-016-0679-7 (PMC5146860; doi:10.1186/s13068-016-0679-7)
Supplement: Supplementary file 2 — Additional file 2: Table S2. Primers used in this study. [file 13068_2016_679_MOESM2_ESM.pdf]

Table S2. Primers used in this study [a].

Primer	Sequence
Cra-F	AGCT***GAATTC***GTGAAACTGGATGAAATCGCTCG
Cra-R	AATT***GGATCC***TTAGCTACGGCTGAGCACGCCGC
Cra-SacI-F	GATC***GAGCTC***GTGAAACTGGATGAAATCGCTCGG
Cra-His-HindIII-R	ACAG***AAGCTT***TTA**GTGATGGTGATGGTGATG**GCTACGGCTGAGCA
pfkB-F(RT)	CAGCACTGGCAATTGGTAACA
pfkB-R(RT)	TTTGGCCTTGCCGCTATT
ppc-F(RT)	CTGTTTGAAACCCTCGATGA
ppc -R(RT)	ACCATCTGTTTGCCCTGAAT
pck-F(RT)	GAACACCCGCGTTTCTTATC
pck -R(RT)	GAAAGCATCAGCAGTCAGGA
mdh-F(RT)	CCCGAAAGCGTGCATTGGTA
mdh-R(RT)	TGGCTGTTTGCCTTTCAG
fumB-F(RT)	CGGTAGATGGCGATGAGTAC
fumB-R(RT)	ACCGAGGGTACGCATTTT
frdA-F(RT)	GGTATGGGTATGGCGCTAAG
frdA-R(RT)	GGTCATCAGGATACCGGAAC
gltA-F(RT)	TTACCCGTCTGTTCCATGCT
gltA-R(RT)	CACGGTGACGAGGATTGTT
icd-F(RT)	AGGTTTATGGTCAGGACG
icd-R(RT)	GCAGGCAGATGTAGAGAT
aceA-F(RT)	GTCGGATATGGGCTACAA
aceA-R(RT)	TTCCTGCTGGTGAGATAC
aceB-F(RT)	GGCAGTGACGATGGATAA
aceB-R(RT)	GTGACCGTTATTGGCTTC
iclR-F(RT)	GGTCAATATGGCGGTGCTT
iclR-R(RT)	CTTCGCTCAGTTGGGCTAAA
aceBp-F	TCGTTAAGCGATTCAGCA
aceBp-R	TGCTGAATCGCTTAACGA
pckp-F	TGGTGAATCGATACTTTA
pckp-R	TAAAGTATCGATTCACCA

[a] Italic and bold bases encode restriction site and underlined bases encode 6 * His tag.
