# Supplementary material for: Succinate production positively correlates with the affinity of the global transcription factor Cra for its effector FBP in Escherichia coli
Source: Biotechnol Biofuels. 2016 Dec 8;9:264. doi: 10.1186/s13068-016-0679-7 (PMC5146860; doi:10.1186/s13068-016-0679-7)
Supplement: Supplementary file 3 — Additional file 3: Table S3. The relationship between succinate production and the affinity of Cra for FBP. [file 13068_2016_679_MOESM3_ESM.pdf]

Table S3. The relationship between succinate production and the affinity of Cra for FBP.

		Affinity	Succinate production
	Pearson correlation	1	-0.894**
**Affinity**	Significance		0.000
	N	37	37
	Pearson correlation	-0.894**	1
**Succinate production**	Significance	0.000	
	N	37	37

**. Significance correlation at 0.01 level.
