# Supplementary figures and images for: Succinate production positively correlates with the affinity of the global transcription factor Cra for its effector FBP in Escherichia coli
Source: Biotechnol Biofuels. 2016 Dec 8;9:264. doi: 10.1186/s13068-016-0679-7 (PMC5146860; doi:10.1186/s13068-016-0679-7)

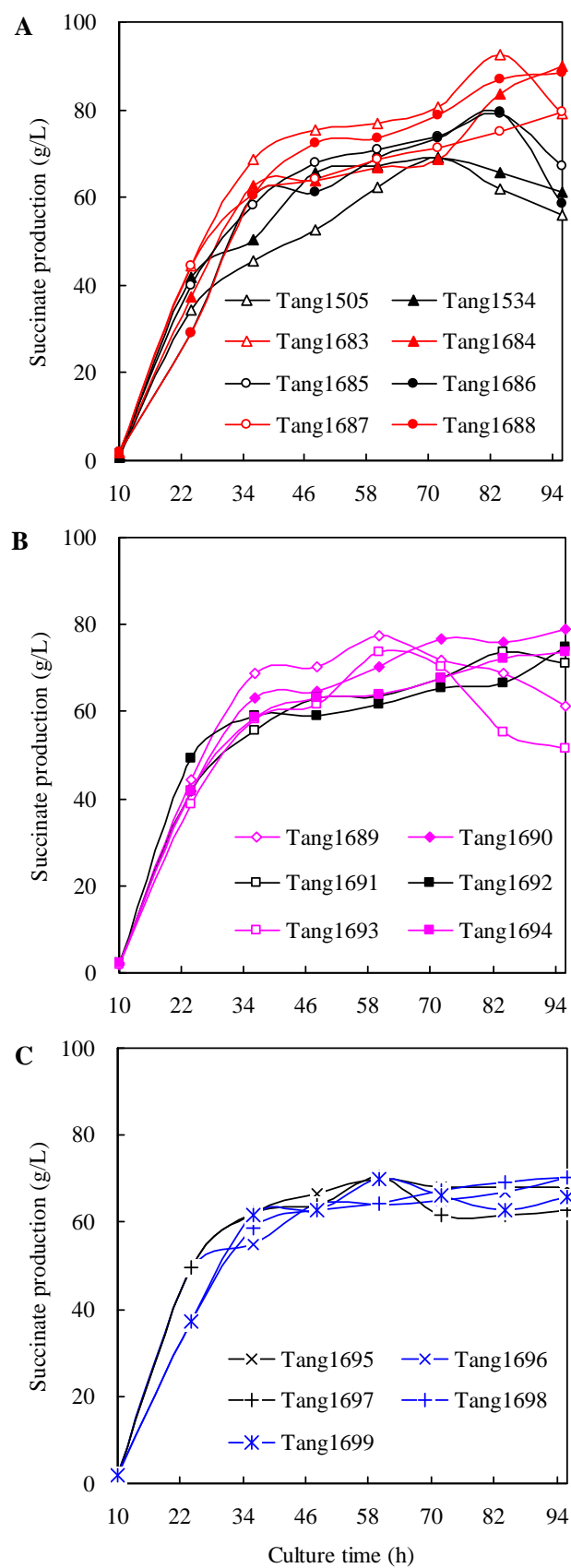

**Figure S1.** Time courses of fed-batch cultures of the 17 three-point mutant strains in 7.5-L bioreactor.

Supplement: Supplementary file 4 — Additional file 4: Figure S1. Time courses of fed-batch cultures of the 17 three-point mutant strains in 7.5-L bioreactor. [file 13068_2016_679_MOESM4_ESM.pdf]
